# Novel Physics-Informed Indicators for Leak Detection in Water Supply Pipelines

**DOI:** 10.3390/s25165069

**Published:** 2025-08-15

**Authors:** Yi Zhang, Suzhen Li

**Affiliations:** 1 College of Civil Engineering, Tongji University, Siping 1239, Shanghai 200092, China; 2 State Key Laboratory of Disaster Reduction in Civil Engineering, Tongji University, Siping 1239, Shanghai 200092, China

**Keywords:** water supply pipeline, leakage noise, physical indicator, leakage detection

## Abstract

**Highlights:**

What are the main findings?

- A robust physics-informed indicator for leak detection in water supply pipelines is proposed, grounded in the physical mechanism of leakage noise sources.
- The proposed physics-informed indicator consistently ranks first in feature importance in both experiment and field testing, showing a clear advantage over conventional statistical features.
- Both the SVM and XGBoost models achieve high recognition accuracy in the experiment and field testing, demonstrating the robustness and generalization capability of the recognition models built on the proposed indicator.

What is the implication of the main finding?

- A robust physics-informed indicator for leak detection in water supply pipelines is proposed, grounded in the physical mechanism of leakage noise sources.
- The proposed physics-informed indicator provides a reliable and interpretable feature for leak detection, with high robustness and strong sensitivity to leakage events.
- The proposed method provides a practical and effective physical feature indicator for complex operating conditions in in-service pipeline networks, demonstrating strong potential for engineering applications.

**Abstract:**

Accurate monitoring of leakage in urban water supply pipelines is crucial for ensuring the safety of residential water usage. This study proposes a robust physical indicator for identifying leaks in urban water pipelines, grounded in the physical background of leakage noise sources. An integral form of the leakage source noise power spectral density is established, and a rigorous theoretical analysis leads to the development of an effective physical indicator. This indicator addresses the limitation of existing leakage detection methods that overly rely on data-driven features. Experiments were conducted to validate the effectiveness and robustness of the proposed indicator. The results show that the leakage detection models trained with physical features achieved recognition accuracies of 99.89% for Support Vector Machine (SVM) and 99.97% for eXtreme Gradient Boosting (XGBoost) in the experiments. In the field test conducted on an in-service water supply pipeline with a total length of 701 m, the recognition accuracies for SVM and XGBoost were 97.92% and 99.31%, respectively.

## 1. Introduction

The water supply network is a crucial infrastructure for the normal functioning of modern cities. With rapid economic development and urban construction, the pace of infrastructure development has accelerated. However, alongside the rapid expansion of the water supply network, leakage accidents caused by pipeline aging, third-party damage, and other factors occur frequently. According to statistics, in 2024, China recorded a total of 3053 underground pipeline damage incidents, a year-on-year increase of 55.4%. Among these, 2419 were leakage-related incidents, accounting for 79.2% of the total, and 2104 were water supply pipeline incidents, accounting for 68.9% of the total [1]. These data highlight that water supply pipelines are the most common type of underground pipeline involved in accidents, and leakage accidents are the most prevalent type of incident. Frequent water supply pipeline accidents not only result in significant resource wastage and disrupt the normal operation of cities, but also pose serious threats to public security and property, leading to severe social impacts. Therefore, timely and effective early warning for water supply pipeline leaks is an urgent issue that needs to be addressed.

With the development of sensor and communication technologies, various leak detection methods based on acoustic emission, fiber optic signals, fluid pressure, and fluid-borne acoustic waves have been extensively studied [2,3,4,5]. Among these methods, acoustic signal detection has gained widespread use in the leak detection field in recent years due to its high sensitivity to leakage events [6,7]. This method is based on turbulence near the leakage hole and the interaction between the fluid inside the pipe and the pipe wall, which generates sound waves that propagate through the internal medium, pipe wall, and soil [8,9]. By analyzing the characteristics of the acoustic signals, the condition of water pipelines can be identified. In the field of acoustic signal technology, depending on the location of signal acquisition, the methods can be classified into solid-borne acoustic wave technology and fluid-borne acoustic wave technology. Solid-borne acoustic wave methods, such as those based on acoustic emission, fiber optics, or accelerometers, capture vibrations in the pipe wall or soil caused by the leakage. In contrast, fluid-borne acoustic wave methods directly capture changes in the fluid waves inside the pipeline caused by the leak. Due to the rapid attenuation and dispersion of sound waves along the pipe wall and soil [10], solid-borne acoustic methods typically have a shorter detection range, usually within a few hundred meters. On the other hand, fluid-borne acoustic wave technology, which is based on internal parameters, has advantages such as high sensitivity and longer detection ranges, and it has been widely applied in pipeline leak monitoring and detection [11].

Currently, the “feature extraction + algorithm identification” data-driven approach is widely applied in the field of acoustic signal leakage detection. In terms of feature extraction, a significant body of research has attempted to extract various statistical features from acoustic signals to distinguish between leakage and non-leakage states in water supply pipelines. In terms of time-domain statistical features, Meng et al. [12] enhanced the accuracy of pipeline leakage detection by combining cumulative value differences, mean value differences, and peak value differences with traditional time-domain features, such as waveform, mean, kurtosis, skewness, and amplitude. Wang et al. [13] used time-domain features of leakage signals for pipeline leakage identification, selecting indicators that do not rely on signal waveform, amplitude, or energy distribution, and demonstrated strong robustness and high recognition accuracy in experimental results. Fabbiano et al. [14] proposed using the RMS value of the signal as a leakage discrimination criterion based on the concept that leakage increases signal energy, observing a linear relationship between the RMS value of the leakage signal and leakage rate. In terms of frequency-domain statistical features, Sitaropoulos et al. [15] employed continuous wavelet transform, power spectral analysis, and frequency band power analysis to identify leakage characteristics, finding that the power in the 150–450 Hz frequency band continuously increased due to leakage. Yazdekhasti et al. [16] used the cross-spectral density between leakage signals as an indicator for leakage detection, which was found to be insensitive to background noise and capable of reflecting leakage size to some extent. In conclusion, the extraction of acoustic signal features is still largely based on statistical features. However, under varying surrounding conditions, pipeline parameters, and operating pressures, these statistical features may not possess the same level of leak detection capability. Their robustness can be called into question [17], which highlights the inherent limitation of statistical features. Therefore, relying solely on statistical features for leak detection inevitably comes with limitations. Current research also faces challenges due to the lack of effective physical features in data analysis, which limits the reliability, applicability, and accuracy of the detection technology.

In terms of algorithm identification, data-driven methods rely on features extracted from historical data and use various machine learning algorithms to analyze data from both leakage and non-leakage conditions for leak detection. Since data-driven methods only require operational data and historical information, they have garnered significant attention in pipeline leakage detection and monitoring, highlighting their promising application potential. Currently, various machine learning algorithms, such as Support Vector Machine (SVM), eXtreme Gradient Boosting (XGBoost), Convolutional Neural Networks (CNN), and Fast Independent Component Analysis (FastICA), are widely applied in leakage detection. For instance, S. El-Zahab et al. [18] compared SVM, Decision Tree (DT), and Naive Bayes (NB) to identify leakage; Shukla et al. [19] developed a CNN-based leakage detection model using a pre-trained AlexNet network. These machine learning and deep learning methods can learn from input features and make effective predictions about the operating conditions of the pipeline.

In conclusion, due to the complexity of the operational conditions of water supply pipelines, relying solely on conventional time-frequency domain statistical features extracted from signals is no longer sufficient to meet high-accuracy leak detection demands in the field of fluid-borne acoustic wave technology. To address these limitations, this study investigates the leak noise source mechanism in rigid water supply pipelines and proposes a physical indicator based on the leakage noise power spectral density. In the algorithm identification section, this paper will use two classical algorithms, Support Vector Machine (SVM) and eXtreme Gradient Boosting (XGBoost), to build a leakage detection model. These two algorithms represent a linear model with good generalization ability and an ensemble learning method with strong fitting capabilities, respectively. By combining different feature sets and evaluating their performance in classification tasks, a leak condition recognition model is constructed.

## 2. Physical Indicator Derived from the Leakage Noise Source Mechanism

This section begins with the theory of aeroacoustics to explore the sound generation mechanism of leakage caused by turbulence from a small hole in a pipeline. A leakage source power spectral density model is established, and through rigorous theoretical derivation, the relationship between the source power spectral density and frequency is derived. Finally, a physical indicator, grounded in the mechanism of leakage noise generation, is proposed.

It is worth noting that pipeline small hole leakage, driven by medium-to-high pressure, is a typical high Reynolds number jet flow problem. Under high Reynolds number conditions, the movement of small- to medium-scale turbulence structures tends toward statistical isotropy, and the statistical characteristics of velocity disturbances no longer depend on a specific direction [20]. Additionally, the jet turbulence generated by the leakage is primarily concentrated in a limited region near the leakage hole, with the spatial scale being much smaller than the wavelength and observation distance. Therefore, it can further be assumed that the turbulent field within this region is homogeneous. The subsequent derivations are based on these two assumptions.

Based on aero-acoustic theory, when a liquid medium is expelled from the leakage hole under the pressure in the pipeline, turbulence is formed near the leakage hole, resulting in leakage noise. The fluctuation equation driven by turbulent stress and the expression of the Lighthill equation are as follows [21]:

(1) $$\frac{1}{c_{0}^{2}}\frac{\partial^{2}p}{\partial t^{2}}-\nabla^{2}p=\frac{\partial^{2}T_{ij}}{\partial x_{i}\partial x_{j}}$$

where p represents the acoustic pressure, $c_{0}$ denotes the velocity of sound in the fluid, *t* is the time variable, $x=\left(x_{1},x_{2},x_{3}\right)$ is typically the spatial coordinate or position vector, i and j represent indices for the directional components, and $T_{ij}$ is the turbulence stress tensor. Generally, the turbulence stress tensor is primarily influenced by the Reynolds stress $\rho u_{i}u_{j}$; thus, Equation (1) can be simplified as:

(2) $$\frac{1}{c_{0}^{2}}\frac{\partial^{2}p}{\partial t^{2}}-\nabla^{2}p=\frac{\partial^{2}\rho_{0}u_{i}u_{j}}{\partial x_{i}\partial x_{j}}$$

where $u_{i},u_{j}$ is the velocity component in the i-direction and j-direction, $\rho_{0}$ represents the fluid density.

### 2.1. Leakage Noise Power Spectral Density

For any given observation point, the corresponding linear solution of pipeline leakage noise can be expressed as:

(3) $$p(x,t)=\rho_{0}\int_{-T}^{T}\int_{V}G(x,t;y,\tau )\frac{\partial u_{i}(y,\tau )u_{j}(y,\tau )}{\partial y_{i}\partial y_{j}}dV(y)d\tau$$

where p(x,t) denotes the leakage noise acoustic pressure at observation position $x=\left(x_{1},x_{2},x_{3}\right)$ and time *t*, G(x,t;y,τ) represents the Green’s function of the pipeline, which describes the acoustic influence propagating from the source point $y=\left(y_{1},y_{2},y_{3}\right)$ at time τ to the observation point $x=\left(x_{1},x_{2},x_{3}\right)$ at time *t*. Taking the Fourier transform on both sides of Equation (3) results in the leakage noise spectrum p(x,ω): Taking the Fourier Transform to obtain the pressure frequency spectrum and $\tilde{p}(x,\omega )$. Based on the power spectral density (PSD) formula $S_{pp}(x,\omega )=E\left[\tilde{p}(x,\omega ){\tilde{p}}^{*}(x,\omega )\right]$, it can be obtained that:

(4) $$S_{pp}(x,\omega )=\rho_{0}^{2}\int_{V}\int_{V}\tilde{G}(x,y,\omega ){\tilde{G}}^{*}\left(x,y^{\prime },\omega \right)S_{u_{i}u_{j}}\left(y,y^{\prime },\omega \right)dV(y)dV\left(y^{\prime }\right)$$

where $S_{u_{i}u_{j}}\left(y,y^{\prime },\omega \right)$ represents the cross-power spectral density of the turbulent velocity.

### 2.2. Physical Indicator

This section will extract universal frequency-domain features from Equation (4). As mentioned earlier, the small hole leakage discussed in this paper is a typical high Reynolds number jet flow. Near the leakage point, the turbulence source term (i.e., the sound source) is assumed to be concentrated in a very small region (on the millimeter scale), and the observation point is typically set several tens of centimeters or even meters away, satisfying the “far-field” condition. Therefore, within the integration region *V*, the Green’s function can be approximated as being insensitive to the spatial variables of the source point:

(5) $$\tilde{G}(x,y,\omega )\approx \tilde{G}\left(x,y_{0},\omega \right),\;\forall y\in V$$

Therefore, the integral expression can be extracted as a constant factor, simplifying to:

(6) $$S_{pp}(\omega )\propto |\tilde{G}(\omega ){|}^{2}\cdot \iint S_{u_{i}u_{j}}\left(y,y^{\prime },\omega \right)dV(y)dV\left(y^{\prime }\right)$$

On the other hand, the isotropy and homogeneity of the turbulent field imply that $S_{u_{i}u_{j}}\left(y,y^{\prime },\omega \right)$ only depends on the relative position vector $r=y-y^{\prime }$ and frequency ω,

(7) $$S_{u_{i}u_{j}}\left(y,y^{\prime },\omega \right)=S_{uu}(r,\omega )$$

Furthermore, by separating the variables, the turbulence energy spectral density can be expressed as the product of the power spectral function $S_{uu}(\omega )$ and a normalized spatial correlation function f(r), yielding:

(8) $$S_{u_{i}u_{j}}\left(y,y^{\prime },\omega \right)=S_{uu}(\omega )\cdot f\left(y-y^{\prime }\right)$$

At this point, the integral result of Equation (6) represents the weighted form of the total turbulence energy. Therefore, the final expression for the leakage sound power spectral density can be simplified to:

(9) $$S_{pp}(\omega )\propto |\tilde{G}(\omega ){|}^{2}\cdot S_{uu}(\omega )$$

This expression indicates that the frequency distribution of the sound pressure power spectral density is jointly determined by the Green’s function and the turbulence velocity spectrum. For the Green’s function, the solution for an infinitely long cylindrical hard-wall pipeline can be expressed as the sum of a finite number of propagating modes and an infinite number of cutoff modes. The Green’s function for the pipeline simplifies to [22]:

(10) $$\hat{G}(x,y,\omega )=\frac{1}{2ik_{0}A_{D}}e^{ik_{0}|x-y|}$$

and

(11) $$|\hat{G}(\omega ){|}^{2}={\left|\frac{1}{2ik_{0}A_{D}}\right|}^{2}=\frac{1}{4k_{0}^{2}A_{D}^{2}}=\frac{c_{0}^{2}}{4A_{D}^{2}\omega^{2}}$$

where $A_{D}$ represents the cross-sectional area of the pipeline, $k_{0}=\omega /c_{0}$ represents the wavenumber. For the turbulence velocity power spectral density at the leakage hole, the von Kármán spectrum is used [23]:

(12) $$S_{uu}(\omega )=\frac{\bar{u^{2}}\Lambda }{\pi U}\cdot \frac{1}{{\left(1+{\left(\frac{\omega \Lambda }{U}\right)}^{2}\right)}^{5/6}}$$

where $\bar{u^{2}}={\left(IU\right)}^{2}$ represents the mean square velocity, which can be determined by setting the turbulence intensity I and combining it with the outlet flow velocity U, Λ is the integration scale (typically the leakage hole diameter). Substituting Equations (11) and (12) into Equation (9):

(13) $$S_{pp}(\omega )\propto |\tilde{G}(\omega ){|}^{2}\cdot S_{u}(\omega )=\frac{c_{0}^{2}}{4A_{D}^{2}\omega^{2}}\cdot \frac{\bar{u^{2}}\Lambda }{\pi U}\cdot \frac{1}{{\left(1+{\left(\frac{\omega \Lambda }{U}\right)}^{2}\right)}^{5/6}}$$

From Equation (13), it can be observed that $S_{pp}(\omega )$ exhibits an approximate power-law dependence on ω, where the exponent is essentially independent of the pipe diameter and leakage orifice parameters. This means that, when transformed into the logarithmic domain, $S_{pp}(\omega )$ will display a relatively stable slope. Such a stable slope can serve as a distinctive indicator of leakage, theoretically offering good robustness against variations in pipeline structure and leakage conditions. The $S_{pp}(\omega )$ will asymptotically follow a function of frequency with a $\omega^{-3.67}$ dependence at slightly higher frequencies. In the log–log scale, the power spectral density of leakage noise will exhibit a linear relationship with frequency. In contrast, under normal operating conditions of a pipeline without leakage, the sound source primarily originates from environmental noise or sensor background noise. Such disturbances generally exhibit a frequency distribution that is uniform or slowly increasing, characteristic of white noise. The power spectral density is essentially independent of frequency, displaying a flat spectral distribution. Even with significant noise interference, its PSD features are concentrated in lower-frequency regions. Based on existing research and engineering practice, the main frequency of leakage noise signals in water supply pipelines is concentrated in the range of 10^1^ Hz to 10^3^ Hz. Therefore, the fitted slope in the log–log domain of the leakage power spectral density within this frequency band (referred to as $s_{psd}$) can be used as the physical indicator for pipeline leakage detection.

**Figure 1:**
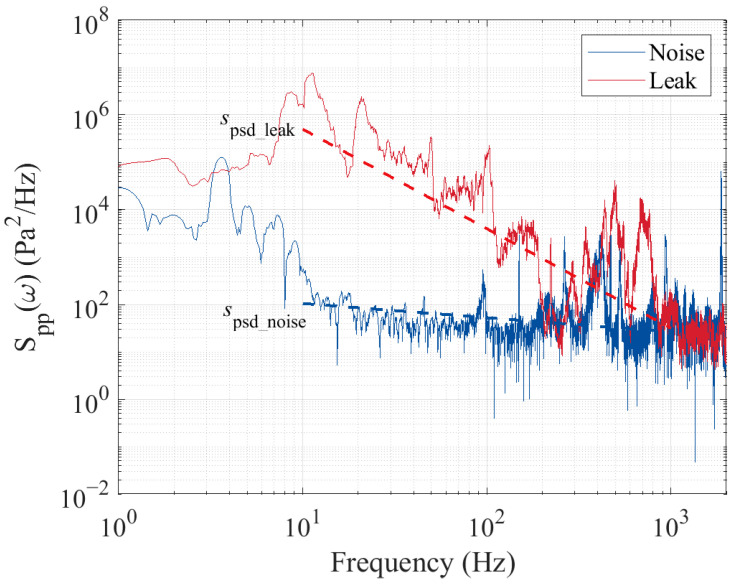
Typical PSD of acoustic signals for leak-free and leak cases.

Figure 1 shows the power spectral density of signals in typical leakage and noise states of a water supply pipeline. It can be observed that, in the log–log scale, the power spectral density of leakage noise exhibits an approximately linear relationship with frequency in the range of 10^1^ Hz to 10^3^ Hz. In contrast, while the power spectral density of background noise also follows a linear relationship with frequency within a certain range, its corresponding gradient tends to flatten.

Combining theoretical analysis with actual measurement results, it is evident that the PSD characteristic exponent $s_{psd}$ in the range of 10^1^ Hz to 10^3^ Hz is a well-defined physical indicator for distinguishing leakage. Further examination of Equation (13) reveals that the characteristic exponent $s_{psd}$ is theoretically independent of parameters such as leakage velocity and hole diameter, and is not affected by signal strength. The integration scale Λ only influences the primary frequency range of the leakage noise. Theoretically, the characteristic exponent $s_{psd}$ demonstrates excellent robustness, making it suitable for leakage detection in water supply pipelines under varying operating conditions.

## 3. Methodology

**Figure 2:**
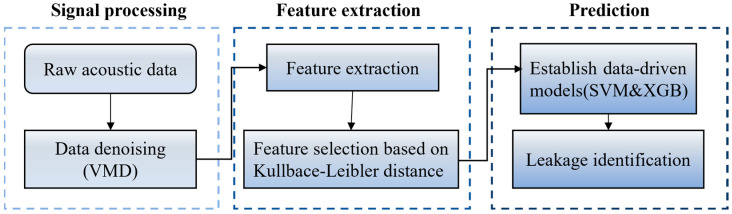
Flowchart of the proposed methodology.

Data-driven machine learning algorithms possess strong pattern recognition capabilities and can automatically learn underlying patterns from large volumes of historical or real-time data. These methods are particularly effective in handling high-dimensional, nonlinear, and non-stationary signals, making them well-suited for complex and dynamic operating conditions. In this study, the proposed physical indicator $s_{psd}$ is integrated with data-driven methods for leakage identification. The overall workflow of the proposed approach is illustrated in Figure 2.

### 3.1. Signal Processing

This study employs an adaptive Variational Mode Decomposition (VMD) method to denoise the raw signals. VMD [24] decomposes the signal into *k* intrinsic mode functions (IMFs) by solving a variational problem that optimizes the central frequency and bandwidth of each component. Denoising effectiveness depends on two factors: (1) selecting the effective IMFs and (2) determining the optimal *k*. For IMF selection, traditional methods rely on empirical parameters [25]. This study uses a correlation analysis, assuming that noise from hydrophones at different locations is uncorrelated, while acoustic leakage signals are correlated. The correlation coefficient between the *k*-th IMF of the detection signal and the reference signal is used, with values $r_{k}=\frac{\sum_{i=1}^{n}\left(u_{k_{i}}-\bar{u}\right)\left(y_{i}-\bar{y}\right)}{\sqrt{\sum_{i=1}^{n}{\left(u_{k_{i}}-\bar{u}\right)}^{2}\sum_{i=1}^{n}{\left(y_{i}-\bar{y}\right)}^{2}}}\geq 0.4$ indicating effective components.

To determine *k*, this study minimizes information entropy *H*(*x*) to avoid over-decomposition or frequency aliasing. Higher entropy indicates greater disorder, with the optimal *k* corresponding to the lowest entropy. The information entropy *H*(*x*) of a one-dimensional random sequence is given by [26]:

(14) $$H(x)=-\overset{n}{\underset{i=1}{\sum }}p\left(x_{i}\right)\log p\left(x_{i}\right)$$

### 3.2. Feature Extraction and Feature Selection Criteria

Extracting effective features enables a targeted representation of pipeline operational state information within the raw data, thereby improving identification performance and reducing training complexity. After denoising the raw acoustic signals, traditional time-domain features, frequency-domain features, and $s_{psd}$ are extracted. The extracted features are shown in Table 1, and features are ranked and selected based on their significance.

In Table 1, $x=\left[x_{1},x_{2},\cdots x_{N}\right]$ represents the time-domain signal of the leakage noise, and the corresponding frequency $f=\left[f_{1},f_{2},\cdots f_{N}\right]$ represents the frequency domain. $X=\left[X_{1},X_{2},\cdots X_{N}\right]$ corresponds to the power spectral density set $\left[P_{xx,1},P_{xx,2},\ldots ,P_{xx,N}\right]$, where $\bar{x}$ is the average value of the signal, and $std(x)=\sqrt{\frac{1}{N}\sum_{i=1}^{N}{\left(x_{i}-\bar{x}\right)}^{2}}$ represents the standard deviation of the signal.

Not all features in Table 1 are relevant to the pipeline’s operational state. Irrelevant features increase training complexity and degrade model performance. To improve feature selection, the Kullback–Leibler (KL) divergence [27] is used. The KL divergence measures the difference between the probability density functions of two random variables. A larger KL divergence indicates better differentiation between feature distributions, making it more effective for distinguishing pipeline conditions. The KL divergence is defined as:

(15) $$d_{kl}=D_{12}+D_{21}$$

(16) $$D_{12}=\sum p\left(s\left|\omega_{1}\right.\right)\log\frac{p\left(s\left|\omega_{1}\right.\right)}{p\left(s\left|\omega_{2}\right.\right)}$$

(17) $$D_{21}=\sum p\left(s\left|\omega_{2}\right.\right)\log\frac{p\left(s\left|\omega_{2}\right.\right)}{p\left(s\left|\omega_{1}\right.\right)}$$

where $\omega_{1}$ and $\omega_{2}$ represent the two different data categories, $p\left(s\left|\omega_{i}\right.\right)$ denotes the conditional probability density function of feature *s* under the i-th data category.

### 3.3. Leak Identification

The selected feature vectors, as described above, will serve as inputs for the data-driven methods. The model training process will establish the mapping relationship between feature states and pipeline operational states. Currently, data-driven machine learning methods are widely used in fault diagnosis and condition recognition, including various models such as neural networks, decision trees, support vector machines, and ensemble learning, especially demonstrating good performance when handling structured features and nonlinear relationships.

For the indicator $s_{psd}$ with physical significance extracted in this study, to achieve efficient identification of leakage and non-leakage conditions, this paper selects two typical supervised learning models—Support Vector Machine (SVM) and Extreme Gradient Boosting (XGBoost)—for classification modeling. As a traditional machine learning algorithm, SVM is based on the concept of constructing an optimal hyperplane and is suitable for small-sample, high-dimensional, and nonlinear classification problems. On the other hand, XGBoost, an ensemble learning algorithm based on gradient-boosted trees, has gained widespread application in recent years due to its significant advantages in feature selection, nonlinear modeling, and generalization ability. The combination of both methods can improve the accuracy and stability of the model while also helping to verify the adaptability and robustness of the physical features across multiple models. This paper will compare the performance of these two algorithms in the application of leakage detection for water supply pipelines. The detailed theoretical background of these data-driven methods is beyond the scope of this paper and can be found in [28,29].

## 4. Experiment Verification and Results

To validate the method developed in Section 3, experiments were carried out to assess the robustness and effectiveness of the proposed indicator. The experiments also aimed to evaluate its recognition performance under various parameters. These tests were designed to simulate different real-world conditions, ensuring that the method is reliable and versatile for practical applications.

### 4.1. Experimental Setup

**Figure 3:**
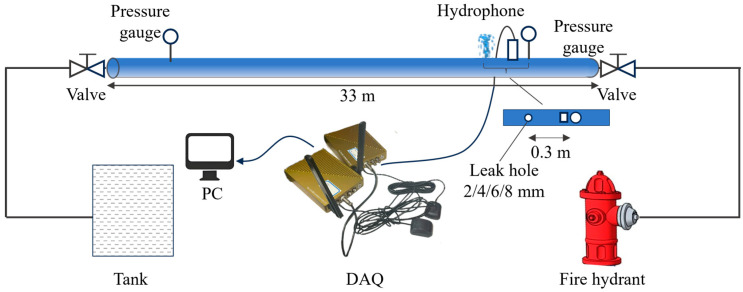
Schematic diagram of the outdoor water supply pipeline layout.

**Figure 4:**
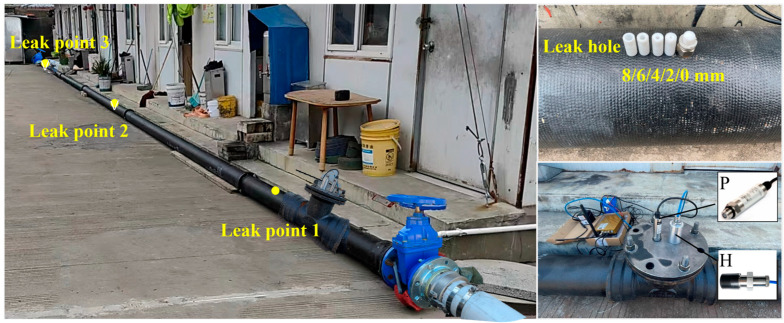
Layout of the experiment.

**Figure 5:**
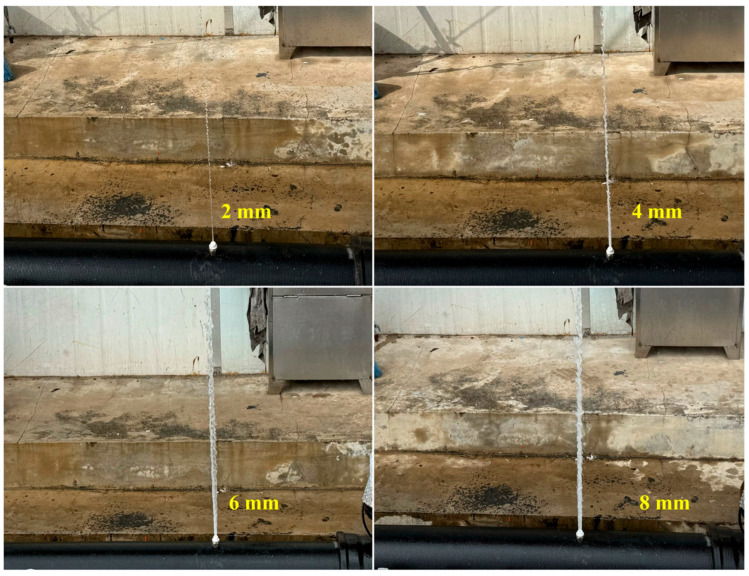
Leakage outlets with different orifice diameters.

The above method is first validated using the experimental testing platform shown in Figure 3 and Figure 4. The experimental pipeline is a 33-m-long ductile iron pipe with a diameter of 150 mm. The upstream of the pipeline is pressurized by an outdoor fire hydrant at the experimental site, and high-frequency pressure sensors are installed at both ends of the test section to monitor the internal pressure in real time. A hydrophone is placed 2 m downstream of the upstream valve to collect the fluid acoustic signals inside the pipe. The specifications of the high-frequency pressure sensors and the hydrophone used in the test are shown in Table 2. The sampling rate for both types of sensors was 4 kHz. Three leakage holes are installed at three positions along the test section (0.3 m, 9.3 m, and 24.3 m downstream of the hydrophone). Each of the three leakage locations is tested with four different hole diameters: 2 mm, 4 mm, 6 mm, and 8 mm. The experimental conditions are listed in Table 3. The pressure supplied by the outdoor fire hydrant to the water inside the pipe is 0.20 MPa. Figure 5 shows the leakage conditions generated by the leakage holes with diameters of 2 mm, 4 mm, 6 mm, and 8 mm.

### 4.2. Feature Selection in the Experiment

**Figure 6:**
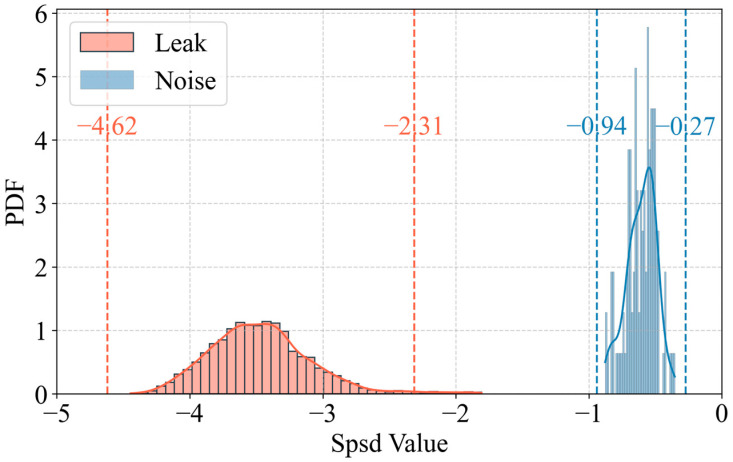
Statistical histogram and probability density function of $s_{psd}$.

Based on the content of Section 2, we extracted the characteristic exponent $s_{psd}$ from the log–log domain PSD of leakage and non-leakage signals in the 10^1^–10^3^ Hz range. The statistical histogram and the probability density function calculated using Kernel Density Estimation (KDE) for $s_{psd}$ are shown in Figure 6, and the 3σ coverage regions are marked in the figure, indicating that 99.7% of the data falls within the ±3σ interval. It can be observed that there is a clear distinction between the distributions of $s_{psd}$ for the two different states. In the leakage state, the distribution of $s_{psd}$ is centered around approximately −3.5, which is in strong agreement with the analytical results from Equation (13). Moreover, the distributions of $s_{psd}$ for leakage and non-leakage states exhibit almost no overlap. Therefore, the proposed physical indicator is capable of distinguishing the operational state of the pipeline system.

**Figure 7:**
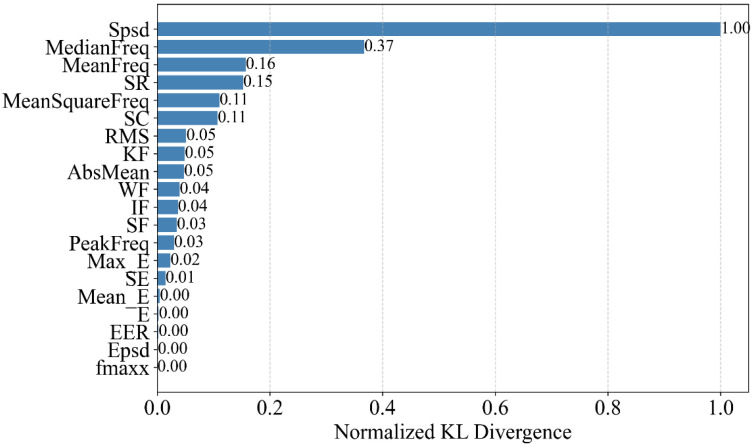
Normalized Kullback–Leibler divergence ranking in the experiment.

The Kullback–Leibler (KL) distance d between the two different events is calculated to further examine the importance ranking of each feature. The normalized value of KL distance d is shown in Figure 7, where $s_{psd}$ ranks first. This indicates that $s_{psd}$, compared to traditional data features, has a better distinguishing ability for pipeline leakage events.

### 4.3. Results of Leak Detection Models in the Experiment

Based on the feature selection results, the top six features are selected as the feature subset to train the SVM and XGBoost models. A total of 70% of the samples are randomly selected for training, and the remaining 30% are used for testing. The parameters for these methods are as follows:

(1) Support Vector Machine (SVM): A linear kernel function is used, and the penalty parameter *c* is set to 47.83.

(2) XGBoost: The tree depth is set to 10, the learning rate is 0.01, and 80% column sampling and 80% sample sampling are employed to enhance generalization ability. The logarithmic loss (logloss) is used as the evaluation metric. To prevent overfitting on the training set through excessive iterations, an early stopping strategy is implemented (early_stopping_rounds = 50). Training is terminated early if the evaluation metric on the validation set does not improve significantly over 50 consecutive iterations, thus improving the model’s generalization ability and training efficiency.

To evaluate the performance of these two methods, the three most important metrics in engineering applications are calculated: accuracy, false positive rate, and false negative rate.

(18) $$Accuracy=\frac{TN+TP}{TN+FN+TP+FP}$$

(19) $$FAR=\frac{FP}{FP+TN}$$

(20) $$FRR=\frac{FN}{FN+TP}$$

where TP is the True Positive, TN is the True Negative, FP is the False Positive, and FN is the False Negative from a confusion matrix.

**Figure 8:**
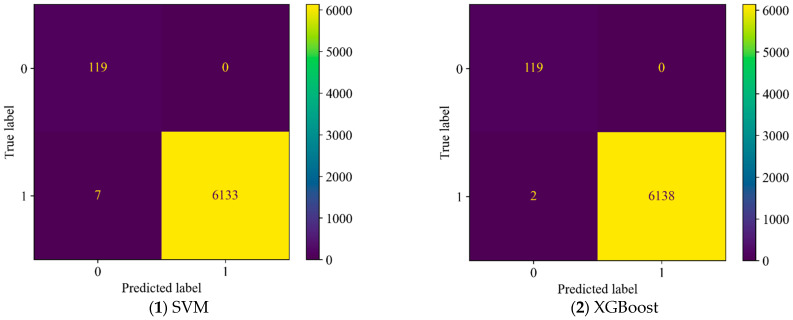
Confusion matrices of both models in the experiment.

Table 4 and Figure 8 show the recognition results for SVM and XGBoost. The results indicate that both SVM and XGBoost perform well in terms of accuracy, false positive rate, and false negative rate. Compared to XGBoost, SVM has a slightly higher false positive rate.

**Figure 9:**
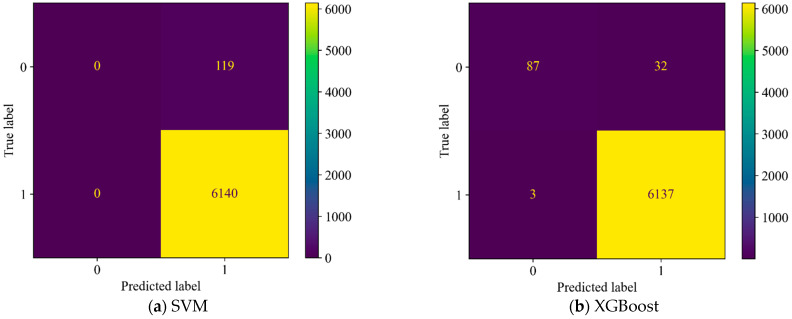
Confusion matrix of both models without $s_{psd}$.

Furthermore, to validate the importance and effectiveness of $s_{psd}$ in leakage detection, we removed $s_{psd}$ and trained and validated the model using the remaining features. The recognition results are shown in Figure 9 and Table 5. From the results, it can be observed that while the accuracy did not decrease significantly, both the false positive rate and false negative rate increased substantially. The false positive rate of the SVM model even reached 100%. Based on the above discussion, it can be concluded that $s_{psd}$ is highly effective in distinguishing between leakage and non-leakage states of the pipeline and has strong irreplaceability within the feature set. The recognition results of the two data-driven models trained with $s_{psd}$ are both satisfactory.

## 5. Field Testing

To validate the generalization ability of the proposed physical feature $s_{psd}$ and evaluate the detection performance of the method incorporating $s_{psd}$ in real-world, in-service water supply pipelines, this study conducted long-distance leakage tests on an operational water supply pipeline.

### 5.1. Overview

**Figure 10:**
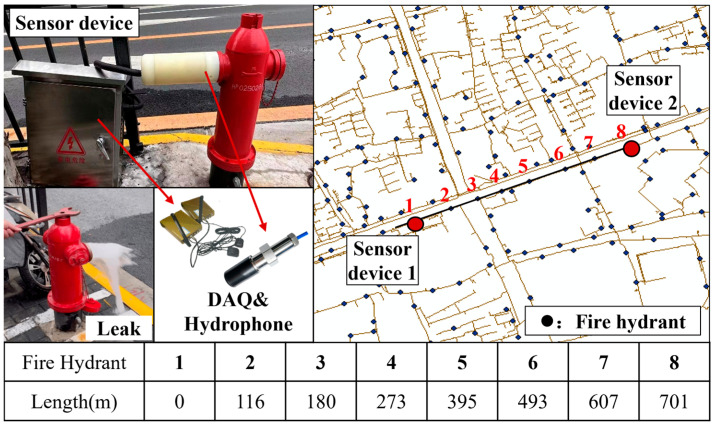
Layout of fire hydrants and sensor equipment on the tested pipeline.

The field test was conducted on an in-service water supply main pipeline in Shanghai, China, as shown in Figure 10. The test section is a DN300 ductile iron pipe with a total length of 701 m, and it includes six fire hydrants. Sensor devices were installed at both ends of the test section (at Fire Hydrants No. 1 and No. 8) on the outlets of the fire hydrants. The internal operating pressure of the pipeline was approximately 0.25 MPa. The devices are equipped with hydrophones (Sinotanden High-tech Technology Co., Ltd., Bejing, China) to capture the fluid acoustic signals inside the pipeline. The collected data were transmitted remotely using wireless data acquisition (DAQ) equipment (Zhongyi Industrial Control Technology Co., Ltd., Shanghai, China), with a sensor sampling rate of 4 kHz. The DAQ units and their power supplies were stored together in a protective box placed beside the fire hydrant. The leakage events were simulated by controlled openings of fire hydrants (No. 2 to No. 7) with 65 mm outlets, each adjusted to approximately half of the maximum valve opening to represent a moderate leak size.

### 5.2. Feature Selection in Field Testing

**Figure 11:**
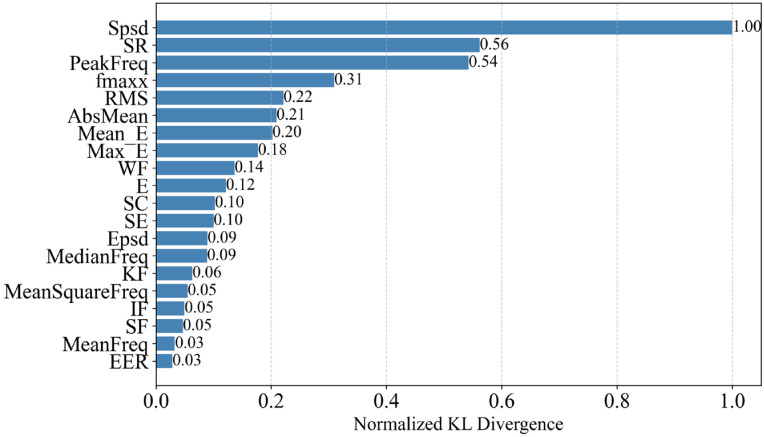
Normalized Kullback–Leibler divergence ranking in the field testing.

Similarly, the Kullback–Leibler (KL) distance for all features in Table 1 (time-domain features, frequency-domain features, and the physical feature $s_{psd}$) was also calculated. The normalized KL distance d for the field test is shown in Figure 11, where $s_{psd}$ ranks first. Comparing this with the ranking from the platform experiments, it can be seen that while the rankings of other data features have changed, $s_{psd}$ consistently ranks first with a significant advantage in both scenarios. This indicates that $s_{psd}$ not only has the best distinguishing ability, but also exhibits very good stability.

### 5.3. Results of Leak Detection Models in Field Testing

Based on the feature selection results, the top seven features were chosen as the feature subset to train the SVM and XGBoost models. A random selection of 70% of the total samples was used for training. The parameters for both models were the same as those used in the platform experiments. After preliminary analysis, it was found that the sensors at both ends of the test section can effectively recognize the simulated leakage conditions for Fire Hydrants No. 2 to No. 7. Therefore, the data from all conditions were used for unified model training and prediction. The prediction results are shown in Table 6.

**Figure 12:**
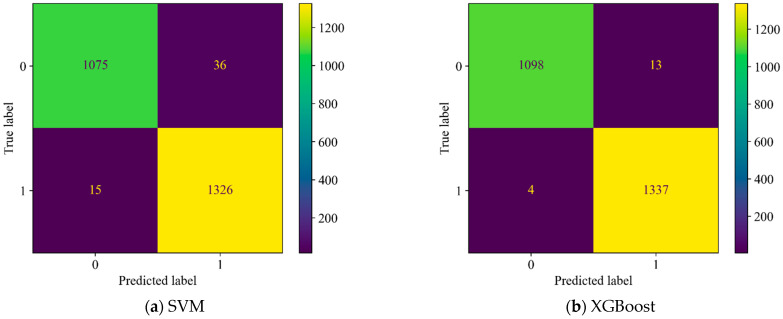
Confusion matrices of both models during field testing.

Figure 12 present the confusion matrices for both models. The results indicate that, in field tests on urban water supply pipelines, the SVM and XGBoost models incorporating the physical feature $s_{psd}$ achieved slightly lower prediction accuracy compared to platform experiments, yet still delivered satisfactory detection performance. Although the decrease in accuracy from platform to field conditions was marginal, both the false acceptance rate (FAR) and false rejection rate (FRR) increased significantly, with FAR showing a particularly notable rise. This may be attributed to the complex conditions inside the pipelines and the presence of substantial background noise in the field environment. As the tested pipeline section was located adjacent to a major urban road, the leakage signals may have been overwhelmed by strong ambient noise, which is likely the primary cause of the increased FRR. In addition, small flow perturbations induced by the complex internal flow conditions of the real pipeline may have contributed to the higher FAR.

## 6. Conclusions

This paper proposes a physics-based leakage detection indicator for water supply pipelines. The indicator is supported by the theoretical model of leakage source power spectral density and has been demonstrated to exhibit good discriminative ability and robustness through experiments. The main conclusions of this paper are as follows:

(1) The turbulence at the leakage hole is considered the primary sound source for leakage noise, and an integral form of the source power spectral density is established. Through analysis, it is concluded that the power spectral density follows an exponential relationship with frequency.
(2) The leakage noise power spectral density $s_{psd}$ will asymptotically follow a $\omega^{-3.67}$ function relationship with frequency in the slightly higher frequency range. In the log–log scale, the power spectral density of leakage noise exhibits a linear relationship with frequency. The characteristic exponent $s_{psd}$ within the frequency range of 10^1^ Hz to 10^3^ Hz, where the main frequency of the water pipeline leakage sound signal is concentrated, is extracted as the physical characteristic for pipeline leakage detection.
(3) The indicator $s_{psd}$ is derived under the assumption of an ideal, infinitely long, rigid pipeline. Therefore, this leakage indicator is applicable only to leakage detection in rigid pipes. Notably, the indicator is minimally affected by parameters such as pipe diameter and leak orifice size, demonstrating strong robustness. The distributions of $s_{psd}$ for leakage/non-leakage and feature ranking results conditions indicate that this indicator can effectively identify leakage events and exhibits good robustness.
(4) Both SVM and XGBoost can be effectively used to establish leakage detection models. In the experiments, the SVM model achieved an accuracy of 99.89%, while XGBoost achieved 99.97%, with XGBoost demonstrating a slight advantage across various metrics. These results indicate that the laboratory experiments strongly validate the physical correctness of the proposed feature $s_{psd}$ as well as its effectiveness in leakage detection.
(5) In the field test, the physical feature $s_{psd}$ is still ranked first in the feature ranking with a substantial lead, demonstrating strong leakage indication capability and good robustness. The prediction accuracies of the SVM and XGBoost models were 97.92% and 99.31%, respectively, slightly lower than those in the platform experiments. However, due to the interference of complex internal flow conditions and strong ambient noise, both the false acceptance rate (FAR) and false rejection rate (FRR) increased, with FAR showing a more pronounced rise. Overall, the leakage detection models based on the physical feature $s_{psd}$ exhibited strong potential for practical engineering applications.
(6) The theoretical and experimental investigation of the physical indicator $s_{psd}$ for leakage requires further refinement. Future research will focus on developing a more detailed physical model of the leakage acoustic source and performing both theoretical and experimental studies on influencing parameters such as leakage orifice geometry, pipeline attachments (e.g., branch pipes, tees, etc.), and more complex pipeline network configurations. These advancements are expected to improve the applicability and robustness of the proposed method in complex and variable real-world engineering environments.

## Figures and Tables

**Table 1:** Features.

Time Domain Feature	Expression	Frequency Domain Feature	Expression	Frequency Domain Feature	Expression
Absolute Mean (‘AbsMean’)	$\frac{1}{N}\sum_{i=1}^{N}\left|x_{i}\right|$	Mean Frequency (‘MeanFreq’)	$\frac{\sum_{i=1}^{N}X_{i}f_{i}}{\sum_{i=1}^{N}X_{i}}$	Waveform Factor (‘WF’)	$\frac{\sqrt{\frac{1}{N}\sum_{i=1}^{N}P_{xx,i}^{2}}}{\frac{1}{N}\sum_{i=1}^{N}P_{xx,i}}$
RMS (‘RMS’)	$\sqrt{\frac{1}{N}\sum_{i=1}^{N}x_{i}^{2}}$	Mean Square Frequency (‘MeanSquareFreq’)	$\frac{\sum_{i=1}^{N}X_{i}f_{i}^{2}}{\sum_{i=1}^{N}X_{i}}$	Spectral Centroid (‘SC’)	$\frac{\sum_{i=1}^{N}f_{i}\cdot P_{xx,i}}{\sum_{i=1}^{N}P_{xx,i}}$
Energy (‘E’)	$\sum_{i=1}^{N}x_{i}^{2}$	Peak Frequency (‘PeakFreq’)	$\begin{array}{l} f_{i}|\\ X_{i}=\max(X) \end{array}$	Spectral Roll-off Frequency (‘SR’)	$\begin{array}{l} f_{k}|\sum_{i=1}^{k}P_{xx,i}\\ \geq \;\mathrm{thre}\cdot \sum_{i=1}^{N}P_{xx,i} \end{array}$
Mean Energy (‘Mean_E’)	$\frac{1}{N}\sum_{i=1}^{N}x_{i}^{2}$	Median Frequency (‘MedianFreq’)	$X_{i}=\frac{1}{2}\sum_{i=1}^{N}X_{i}$	Spectral Entropy) (‘SE’)	$-\sum_{i=1}^{N}P_{xx,i}\cdot \log P_{xx,i}$
Maximum Instantaneous Energy (‘Max_E’)	$\underset{i}{\max}\left(x_{i}^{2}\right)$	Peak PSD Frequency (‘fmaxx’)	$\begin{array}{l} f_{k}|\\ P_{xx,k}=\max P_{xx} \end{array}$	Energy-to-Entropy Ratio (‘EER’)	$\frac{E}{SE}$
Kurtosis Factor (‘KF’)	$\frac{1}{N}\sum_{i=1}^{N}{\left(\frac{x_{i}-\bar{x}}{std(x)}\right)}^{4}$	Energy (‘Epsd’)	$\sum_{i=1}^{N}P_{xx,i}^{2}$	$s_{psd}$	
Skewness Factor (‘SF’)	$\frac{1}{N}\sum_{i=1}^{N}{\left(\frac{x_{i}-\bar{x}}{std(x)}\right)}^{3}$	Impulse Factor (‘IF’)	$\frac{\max\left(P_{xx}\right)}{\frac{1}{N}\sum_{i=1}^{N}P_{xx,i}}$		

**Table 2:** Sensor specifications.

Hydrophone	Parameter	Sensitivity @1 kHz	Frequency Range	Preamplifier Gain
	Value	−156 dB V/μPa	1 Hz–2 KHz	40 dB
High- Frequency Pressure Sensor	Parameter	Measurement Range	Frequency Range	Accuracy
	Value	0–3 MPa	0–2 KHz	±0.2% FS

**Table 3:** Leakage conditions.

Variable	Value
Leak position (Distance from the hydrophone)	0.3 m, 9.3 m, 24.3 m
leakage diameters 2a	2 mm, 4 mm, 6 mm, 8 mm

**Table 4:** Identification results in the experiment.

Model	Accuracy	FAR	FRR
SVM	99.89%	0%	0.11%
XGBoost	99.97%	0%	0.03%

**Table 5:** Identification results without $s_{psd}$ in the experiment.

Model	Accuracy	FAR	FRR
SVM	98.10%	100%	0%
XGBoost	99.44%	26.89%	0.05%

**Table 6:** Identification results in field testing.

Model	Accuracy	FAR	FRR
SVM	97.92%	3.24%	1.12%
XGBoost	99.31%	1.17%	0.30%

## Data Availability

The data that support the findings of this study are available from the corresponding author upon reasonable request.
